# Exome sequencing of an adolescent with nonalcoholic fatty liver disease identifies a clinically actionable case of Wilson disease

**DOI:** 10.1101/mcs.a003087

**Published:** 2018-10

**Authors:** Julia Wattacheril, Patrick R. Shea, Saeed Mohammad, Cynthia Behling, Vimla Aggarwal, Laura A. Wilson, Katherine P. Yates, Joy Ito, Mark Fishbein, Nicholas Stong, Joel E. Lavine, David B. Goldstein

**Affiliations:** 1 Department of Medicine, Center for Liver Disease and Transplantation, Columbia University College of Physicians and Surgeons, New York Presbyterian Hospital, New York, New York 10032, USA;; 2 Institute of Genomic Medicine, Columbia University College of Physicians and Surgeons, New York, New York 10032, USA;; 3 Department of Pediatrics, Northwestern University Feinberg School of Medicine, Ann and Robert Lurie Children's Hospital of Chicago, Chicago, Illinois 60611, USA;; 4 Department of Pathology, University of California, San Diego, La Jolla, California 90293, USA;; 5 Department of Pathology and Cell Biology, Institute of Genomic Medicine, Columbia University College of Physicians and Surgeons, New York, New York 10032, USA;; 6 Department of Epidemiology, Johns Hopkins University, Baltimore, Maryland 21218, USA;; 7 Ann and Robert Lurie Children's Hospital of Chicago, Chicago, Illinois 60611, USA;; 8 Department of Pediatrics, Columbia University College of Physicians and Surgeons, Children's Hospital of New York, New York, New York 10032, USA

**Keywords:** increased urinary copper concentration

## Abstract

Diagnostic whole-exome sequencing has proven highly successful in a range of rare diseases, particularly early-onset genetic conditions. In more common conditions, however, exome sequencing for diagnostic purposes remains the exception. Here we describe a patient initially diagnosed with a common, complex liver disease, nonalcoholic fatty liver disease (NAFLD), who was determined to have Wilson disease (WD) upon research-related exome sequencing. The patient presented as a 14.5-yr-old adolescent with chronically elevated aminotransferases, normal ceruloplasmin, and histologic examination consistent with NAFLD with advanced fibrosis. He was enrolled in a large longitudinal study of patients with NAFLD and was found to have WD by exome sequencing performed 4 yr later. This new diagnosis, confirmed clinically by 24 h urine copper quantification, led to a change in the therapy from lifestyle counseling to directed treatment with d-penicillamine, a copper chelating agent. In this case, the likelihood of making the correct diagnosis and thereby choosing the appropriate treatment was increased by exome sequencing and careful interpretation. This example illustrates the utility of exome sequencing diagnostically in more common conditions not currently considered as targets for genome-wide evaluation and adds to a growing body of evidence that patients diagnosed with more common conditions often in fact have rarer genetically determined syndromes that have escaped clinical detection.

## INTRODUCTION

The traditional diagnostic workup for children with abnormal liver enzymes includes a review of relevant historical factors and physical findings, serological examination, imaging, and in some cases, histopathologic examination (Vos et al. 2017). Despite this diagnostic algorithm, distinguishing rare monogenic diseases from more common complex disease can be difficult. One example of this overlap of clinical findings includes nonalcoholic fatty liver disease (NAFLD) and Wilson disease (WD). NAFLD is the most common liver disease globally (Diehl and Day 2017). Establishing the diagnosis of NAFLD requires exclusion of significant alcohol intake as well as secondary causes of fat accumulation in the liver (hepatic steatosis). The prevalence of hepatic steatosis in obese children has been estimated to be as high as 38% (Schwimmer et al. 2006). NAFLD is associated with obesity, overweight, and the metabolic syndrome. A clinical phenotype of interest for liver-related outcomes is nonalcoholic steatohepatitis (NASH), given its association with liver fibrosis and cirrhosis. Although the diagnostic workup of NAFLD includes serologic screening to exclude other chronic liver diseases, a definitive diagnosis of NASH requires a liver biopsy. The current management of NAFLD includes lifestyle modification to achieve at least 7%–10% weight loss and risk factor modification for comorbid diseases like diabetes and dyslipidemia. Given potential for the development of end stage liver disease and associated mortality, many pharmacologic treatments are in various stages of development for NASH.

One rare liver disease included in the differential diagnosis for NAFLD is WD (also known as hepatolenticular degeneration), an autosomal recessive genetic disorder that leads to copper accumulation in the liver. Screening involves assessment of serum ceruloplasmin, the main copper carrying protein in the blood. Serum ceruloplasmin alone has a low positive predictive value in patients undergoing a diagnostic workup for chronic liver disease. Although normal ceruloplasmin does not exclude the diagnosis, additional evaluation is determined by clinical suspicion in accordance with guidelines (Roberts et al. 2008). Low ceruloplasmin levels have been associated with alcohol and viral liver disease as well as malabsorptive conditions. Whereas a very low serum ceruloplasmin has a higher predictive value for WD than values near the lower limit of normal, normal or elevated ceruloplasmin levels can be seen in patients with WD. Only approximately one-half of patients ultimately diagnosed with WD present classically with disease onset at age 5–35, decreased serum ceruloplasmin, and detectable Kayser–Fleischer rings (copper deposition within Descemet's membrane of the cornea) (Steindl et al. 1997). Additional diagnostic workup in suspected cases includes urinary copper quantification and, in some cases, liver biopsy to establish the hepatic copper concentration by weight. Staining for copper within the liver is often not reliable and failure to detect hepatocellular copper histochemically is particularly common in children presenting with Wilson disease (Jain et al. 1978; MacSween et al. 1979). The histology of WD and NAFLD/NASH can be indistinguishable. Both may show steatosis, ballooned hepatocytes, glycogenated nuclei, and Mallory–Denk bodies. All of these features are much more commonly observed in NAFLD. However, the importance of an accurate diagnosis of WD cannot be overstated: Untreated, WD is universally fatal (Stremmel et al. 1991; Merle et al. 2007). Effective treatment does exist, however, in the form of copper chelating agents. These agents serve to remove or detoxify the tissue copper that has accumulated and prevent reaccumulation. With lifetime chelation in those with chronic disease or liver transplantation in acute liver failure or decompensated liver disease, long-term survival is both expected and observed (Dhawan et al. 2005; Arnon et al. 2011).

Unlike NAFLD which affects ∼25% of the world's population, ∼1 in 30,000 individuals worldwide have WD (Huster 2010). WD is caused by mutations in the gene *ATP7B,* which encodes a copper-transporting P-type ATPase. Loss-of-function mutations in *ATP7B* are associated with progressive liver disease due to copper accumulation in hepatocytes. Consistent with that role in copper metabolism and tissue damage due to copper accumulation, patients most often present with liver disease, which may range from asymptomatic elevations in liver biochemistry to chronic hepatitis with progressive fibrosis to fulminant hepatic failure. Neurologic manifestations alone (dysarthria, ataxia, dystonia, tremor, Parkinsonism) also can come to clinical attention, usually in mid–late-teen or early-adult presentations. Affected individuals may also have neuropsychiatric findings and deposition of copper in the basal ganglia.

Diagnostic whole-exome sequencing has proven highly successful in a range of rare diseases, particularly early-onset genetic conditions. The utility of exome sequencing for diagnostic purposes in common conditions is not well established given cost relative to unproven benefit for screening purposes. Here, we will provide a clear illustration of such utility. As part of a research study of 238 pediatric patients diagnosed with NAFLD, including many with advanced fibrosis, we identified a secure genetic diagnosis of WD resulting in a rapid and potentially lifesaving change in management.

## RESULTS

### Clinical Presentation and Family History

The patient presented in September 2012 at age 14.5 yr for elevated liver enzymes of 6 months’ duration. He reported abdominal pain and nausea. No neurologic or ophthalmologic symptoms were reported. Medications included montelukast for asthma. Family history was positive for dyslipidemia. The patient reported Polish ancestry. BMI was at the 87th percentile (overweight). Blood pressure was mildly elevated. Physical exam revealed hepatomegaly to 14 cm. No ophthalmologic or neurologic signs were noted, including Kayser–Fleischer rings (slit lamp exam not performed). Screening labs obtained according to American Association of the Study of Liver Diseases (AASLD) guidance (Chalasani et al. 2018) revealed a mildly elevated alanine aminotransferase (ALT) of 50 (2–30) IU/l, aspartate aminotransferase (AST) 32 (18–67) IU/l, alkaline phosphatase (AP) 253 (74–318) U/l, total bilirubin 0.4 (0.2–1) mg/dl, albumin 4.5 (3.6–4.7) g/dl, PT 11.1 (9.3–12) sec, INR 1.1, an elevated fasting insulin of 25.43 (normal 2–17) µIU/ml, fasting glucose 91 (70–99) mg/dl, hemoglobin (Hgb) A1c 5.2 (4.1%–5.8%), total cholesterol of 233 (125–170) mg/dl, and normal thyroid studies. Serum ceruloplasmin was normal at 26 (20–50) mg/dl; therefore, a 24-h collection for urinary copper was not performed at that time. Screening tests for other forms of chronic liver disease included viral hepatitides, alpha-1 antitrypsin deficiency, autoimmune hepatitis, and iron panel were unremarkable. Slit lamp exam was not performed given very limited clinical suspicion for Wilson disease on initial presentation. The patient was counseled regarding lifestyle changes for weight loss and referred to the pediatric NASH clinic for follow up in 6 mo.

At follow-up, he complained of headaches and continued to have elevated blood pressure. His physical exam was otherwise unremarkable. Further dietary and physical activity recommendations were given, and a liver biopsy was recommended if repeat liver biochemistry remained elevated. At his next visit, a weight gain of 6.2 kg was noted over the prior 12 mo, and laboratory reassessment revealed ALT 96 U/l, AST 47 U/l, albumin 4.4 g/dl, total bilirubin 0.2 mg/dl, AP 264 U/l (53–223), fasting insulin 21.28 µIU/ml, fasting glucose 106 mg/dl, Hgb A1c 5.0%, and total cholesterol 226 mg/dl.

**Figure 1.**
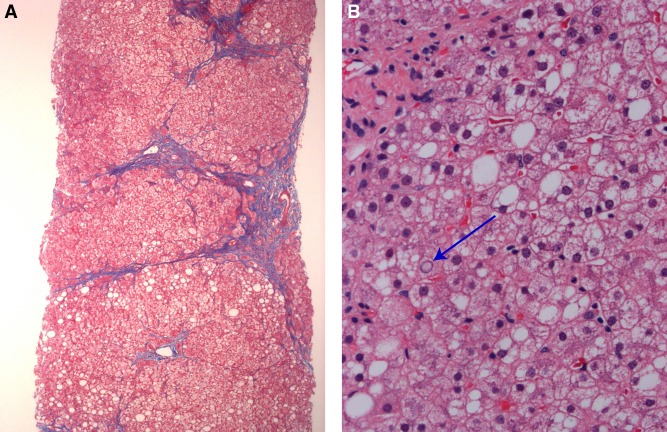
Liver biopsy showed (*A*) focal bridging and perisinusoidal fibrosis (trichrome stain) and (*B*) steatosis with glycogen nuclei (blue arrow; hematoxylin and eosin stain).

Histopathology from a liver biopsy obtained in October of 2013 revealed a vaguely nodular architecture (Fig. 1). The portal areas contained well-preserved bile ducts and patent, unremarkable portal vessels. Two portal areas were expanded by mild chronic lymphocytic infiltrate composed mostly of small mature lymphocytes without interface activity. The hepatocytes were characterized by minimal steatosis (<5%), glycogenated nuclei, and feathery degeneration and a few were ballooned. No Mallory bodies or periodic acid–Schiff (PAS)-D positive globules were identified. The central areas were histologically unremarkable. Trichrome stain revealed periportal fibrosis with multiple bridges and incomplete nodule formation, corresponding to stage 3 fibrosis. The biopsy was reviewed in the context of the Nonalcoholic Steatohepatitis Clinical Research Network (NASH-CRN) by pathologists who were blinded to clinical or demographic information about the case. At this review, the biopsy received a NAFLD activity score (NAS, sum of steatosis grade, lobular inflammation grade, and ballooning grade) of 1.

He remained asymptomatic and presented for clinical follow-up with continued lifestyle changes. He had no subjective complaints or new physical findings. Laboratory assessments for the next four years revealed persistently elevated ALT and AST to 100–255 U/l and 45–107 U/l, respectively. Referral was made to preventative cardiology for dyslipidemia and he was started on omega-3 fatty acids.

Two years after enrollment, an ancillary study was initiated to investigate the relationship of rare variants to advanced fibrosis in pediatric NAFLD and the patient's DNA was included in the cohort (*n* = 238) acquired for exome sequencing.

### Genomic Analysis

Analysis of whole-exome sequencing data for the patient indicated that he was homozygous for a well-known pathogenic missense substitution (NM_000053.3:c.3207C>A p.(His1069Gln)) in the copper transport gene *ATP7B*, located on the long arm of Chromosome 13 (Table 1). Loss-of-function mutations in *ATP7B* are associated with WD. *ATP7B* is expressed primarily in the liver and in normal hepatocytes where it localizes to the *trans*-Golgi network. The ATP7B protein contains six copper binding motifs and a P-type ATPase domain that is conserved among copper transport proteins. ATP7B normally functions as an efflux pump that transports copper from the liver to ceruloplasmin. However, increased intracellular levels of copper within the hepatocyte leads to a change in the distribution of ATP7B to vesicular compartments close to the bile canaliculus to allow excretion of copper to bile. Missense substitutions at codon 1069 have been found to disrupt the catalytic activity of the P-type ATPase domain (Tsivkovskii et al. 2003) and result in retention of the ATP7B protein within the endoplasmic reticulum because of unstable folding (Payne et al. 1998). Homozygosity for the 1069Gln allele is the most common cause of WD among patients of European ancestry (Thomas et al. 1995; Duc et al. 1998; Payne et al. 1998), occurring at a frequency of 1 in 625. As for known variants associated with NAFLD, the patient was homozygous for the wild-type I148 allele for *PNPLA3*.

**Table 1.** *ATP7B* variant information

Gene	Chromosome	HGVS DNA reference	HGVS protein reference	Variant type	Predicted effect	dbSNP ID	Genotype	ClinVar ID
*ATP7B*	13q14.3	NM_000053.3	NP_000044.2	Substitution	His1069Gln	rs76151636	Homozygous	3848

### Communication of Genetic Results and Management Changes

Because of the research context for which the DNA sample was obtained, appropriate permissions were needed from the NASH-CRN Data Coordinating Center (DCC) and local Institutional Review Board prior to identification of the subject and communication to the site principal investigator and treating physician. In particular, the consent and assent for the study did not include return of results, with an exception for very rare circumstances impacting health, as in this case. All of these communications occurred within 6 calendar days (4 business days) of the study team notifying the DCC. Clinical confirmation was obtained with repeat ceruloplasmin of 12.8 mg/dl and 24-h urinary copper, which was elevated at 16.3 mcg/dl (normal 0.2–8 mcg/dl). Confirmation of genetic findings by a CLIA-approved laboratory has confirmed homozygosity for the missense substitution found by exome sequencing. The patient was started on d-penicillamine, which has been tolerated without difficulty and with improvement in his aminotransferases. An asymptomatic brother with normal liver enzymes is undergoing evaluation.

## DISCUSSION

When WD is strongly suspected, clinical genetic testing of *ATP7B* is indicated. WD, however, like many other rare diseases, can be difficult to recognize clinically. In this case, the patient was diagnosed with NAFLD with appropriate phenotyping; however it does not appear that a diagnosis of WD would have been arrived at clinically even with histologic evaluation given identical features in both diseases. This case emphasizes the importance of a heightened clinical suspicion of WD in the diagnostic evaluation of NAFLD, especially in children and adolescents with evidence of advanced fibrosis. However, given the absence of distinguishing histologic features, careful attention must be paid to exclude WD by laboratory and clinical findings, as was done in this case. To our knowledge, this is the first diagnosis of an isolated case of WD through exome sequencing in a longitudinal cohort of NAFLD that resulted in the treatment at a critical stage of liver disease. Even with advanced fibrosis, adherence to chelation therapy will likely result in stability of his disease and regression of fibrosis. Clinical improvement in advanced liver disease is typically observed in the first two to six months of therapy with reversibility of even advanced fibrosis or cirrhosis following prolonged treatment. This outcome would not be expected with lifestyle changes and weight loss alone, as would be recommended (and likely beneficial) for the treatment of NAFLD. Untreated, progression of disease with decompensation and death or need for transplant would be expected. This case adds to a growing number of examples in which exome sequencing provides diagnostic and rapid treatment with clinical benefit that would not have otherwise been obtained (Choi et al. 2009; Bainbridge et al. 2011; Worthey et al. 2011; Johnson et al. 2012; Bearden et al. 2014; Canna et al. 2014; Foley et al. 2014; Iglesias et al. 2014; Romberg et al. 2014; Petrovski et al. 2015; Valencia et al. 2015; Trujillano et al. 2017; Vissers et al. 2017). Further, exome sequencing allowed us to screen the asymptomatic at-risk sibling. The rapidity of notification of the center and patient was facilitated by a network consent that permitted return of results in very rare situations that would provide important health information.

It remains unclear what proportion of patients with serious genetic diseases would derive such clear clinical benefit from exome sequencing. In contrast to unresolved childhood diseases where a genetic cause is suspected and subsequent testing is pursued, our patient presented with findings that met clinical criteria for a very common, complex and heterogeneous disease, NAFLD. Exome sequencing was not planned for clinical purposes and the correct diagnosis was obtained only because the patient happened to be included in a research study designed not for clinical diagnostics but for gene discovery for a distinct condition. This highlights a gap in the clinical application of diagnostic sequencing and suggests clinical value in its deployment in more common conditions. Other relatively common conditions likely to benefit from more widespread genetic evaluations including chronic kidney disease and dyslipidemia (Davis et al. 2017; Lata et al. 2017). Given known heterogeneity present in hepatic steatosis, especially within the pediatric population, it is now a priority to determine what proportion of patients diagnosed with NAFLD may rather have unrecognized syndromes.

## MATERIALS AND METHODS

### Sequencing and Analysis

High-molecular-weight genomic DNA was isolated from the patients’ peripheral blood sample using the PerkinElmer Chemagen automated nucleic acid purification system. Exome enrichment was performed using the NimbleGen SeqCap EZ Exome v3 capture kit according to manufacturer's instructions. Exome sequencing was performed on the Illumina HiSeq2500 System using standard protocols to more than 93× autosomal coverage with 98.6% of the consensus coding sequence (CCDS) regions covered at least 5×. After removal of duplicate sequencing reads using picard-tools-1.59, the remaining reads were aligned to Build 37 of the Human genome reference sequence using BWA version 0.5.10. Variant calling and annotation were performed using GenomeAnalysisToolKit version 1.6-11 and SnpEff version 3.3. Sequence data from 11,090 population control samples obtained from studies unrelated to liver disease, as well as aggregate data from Exome Variant Server (EVS) and Exome Aggregate Consortium (ExAC release 0.3), were used as control samples to define minor allele frequency thresholds. Sequence data was analyzed using a modified version of our previously described trio sequencing protocols (Zhu et al. 2015) designed for use in nontrio data. Qualifying variants with >10× sequencing coverage were required to have <1% minor allele frequency among control samples both external and in-house and have a predicted functional impact based on their SnpEff annotation. Variants were then compared to known or likely pathogenic mutations present in the Human Gene Mutation Database (HGMD) or NCBI ClinVar databases.

## ADDITIONAL INFORMATION

### Data Deposition and Access

Information for the His1069Gln variant described in this report has been submitted to the National Center for Biotechnology Information ClinVar database (https://www.ncbi.nlm.nih.gov/clinvar) under the accession number SCV000778805.1. Exome data has been submitted to dbGaP (https://www.ncbi.nlm.nih.gov/gap); the accession number is pending.

### Ethics Statement

The Institutional Review Boards of Columbia University College of Physicians and Surgeons and the Ann and Robert H. Lurie Children's Hospital of Chicago approved this research protocol (2010-14067) Nonalcoholic Fatty Liver Disease (NAFLD) Pediatric Database 2 (Clinical Center: Nonalcoholic Steatohepatitis Clinical Research Network [NASH CRN]). Informed consent was received from the patient and parent at the time of study enrollment and from the patient for publication.

### Acknowledgments

We thank members of the National Institute of Diabetes and Digestive and Kidney Diseases (NIDDK), Nonalcoholic Steatohepatitis Clinical Research Network (NASH CRN), Data Coordinating Center (DCC) in addition to the Ann and Robert H. Lurie Children's Hospital of Chicago Institutional Review Board for their rapid efforts to help establish contact with the patient.

### Author Contributions

Patient recruitment and phenotyping was done by S.M., M.F., J.I., C.B., L.A.W., K.P.Y., and J.W. Sequence data was analyzed and interpreted by P.S., V.A., N.S., and D.B.G. The manuscript was written by J.W., S.M., P.S., C.B., L.A.W., J.E.L., and D.B.G. All authors contributed to reviewing the final draft.

### Funding

We would also like to acknowledge G. Mani Subramanian and Robert P. Myers of Gilead Sciences, Inc. for providing funding for the sequencing of this specimen as part of a larger proposal.

### Competing Interest Statement

The authors have declared no competing interest.

### Referees

Peter Ferenci

Anonymous

Anonymous

## Supplementary Material

Supplemental Material
