# Supplementary material for: Exome sequencing of an adolescent with nonalcoholic fatty liver disease identifies a clinically actionable case of Wilson disease
Source: Cold Spring Harb Mol Case Stud. 2018 Oct;4(5):a003087. doi: 10.1101/mcs.a003087 (PMC6169823; doi:10.1101/mcs.a003087)
Supplement: Supplemental Material [file supp_4_5_a003087__index.html]

Supplemental Material 

# Exome sequencing of an adolescent with nonalcoholic fatty liver disease identifies a clinically actionable case of Wilson disease

## Supplemental Material

- Supplemental\_Table.xlsx
